# Articulation-based sound perception in verbal repetition: a functional NIRS study

**DOI:** 10.3389/fnhum.2013.00540

**Published:** 2013-09-05

**Authors:** Sejin Yoo, Kyoung-Min Lee

**Affiliations:** ^1^R&D Team, Health and Medical Equipment Business, Samsung ElectronicsSuwon, South Korea; ^2^Interdisciplinary Program in Cognitive Science, Seoul National UniversitySeoul, South Korea; ^3^Department of Neurology, Seoul National UniversitySeoul, South Korea

**Keywords:** verbal repetition, inferior frontal gyrus, articulation-based codes, sound perception, functional near-infrared spectroscopy, hemoglobin concentration, sensorimotor integration

## Abstract

Verbal repetition is a fundamental language capacity where listening and speaking are inextricably coupled with each other. We have recently reported that the left inferior frontal gyrus (IFG) harbors articulation-based codes, as evidenced by activation during repetition of meaningless speech sounds, i.e., pseudowords. In this study, we aimed at confirming this finding and further investigating the possibility that sound perception as well as articulation is subserved by neural circuits in this region. Using functional near-infrared spectroscopy (fNIRS), we monitored changes of hemoglobin (Hb) concentration at IFG bilaterally, while subjects verbally repeated pseudowords and words. The results revealed that the proportion of oxygenated hemoglobin (O_2_Hb) over total Hb was significantly higher at the left IFG during repetition of pseudowords than that of words, replicating the observation by functional MRI and indicating that the region processes articulatory codes for verbal repetition. More importantly for this study, hemodynamic modulations were observed at both IFG during passive listening without repetition to various sounds, including natural environmental sounds, animal vocalizations, and human non-speech sounds. Furthermore, the O_2_Hb concentration increased at the left IFG but decreased at the right IFG for both speech and non-speech sounds. These findings suggest that both speech and non-speech sounds may be processed and maintained by a neural mechanism for sensorimotor integration using articulatory codes at the left IFG.

## Introduction

Verbal repetition is a sort of vocal imitation frequently used for learning languages. The imitation basically indicates sound mimicking without articulating sounds phonetically and phonologically. However, at a certain point of that learning, the imitation is turned into a specific linguistic process, i.e., speech processing that is dependent on limited sounds in a specific phonetic domain (Kuhl, 2004). The language-specific sound learning happens in a continuous manner, and thus it is in general not easy to specify how sounds become speech by learning. Furthermore, sounds processing and speech processing have much in common in terms of neural circuitries (Koelsch et al., 2009), which makes it more difficult to study the difference between sounds and speech.

The categorical perception (Liberman et al., 1957) can provide a helpful insight on solving the problem. Speech sounds are not so different from other sounds such as animal vocalizations and environmental sounds when acoustic sounds are processed along central auditory pathways from outer ears to auditory cortex (Malmierca and Hackett, 2010). However, the situation changes when the incoming signals arrive at the auditory cortex and some higher cortical regions, where the speech sound is perceived not only by its physical properties, but also by various linguistic features. In this sense, it is worthy to note how the brain extracts and deals with the linguistic information embedded on speech sounds. That is, it is important to know speech codes generated and maintained by the brain.

The neuropsychological theories of speech perception suggest at least two kinds of speech codes: acoustic and articulatory codes. The former assumes that speech sounds may be encoded with their acoustic characteristics (Stevens and Blumstein, 1981; Massaro, 1987; Goldinger, 1997; Johnson, 1997; Coleman, 1998), in which neural activities representing speech sounds are more likely to be directly modulated by frequency and duration of sound waves. However, the latter regards speech perception as a process in an articulatory domain, not in an acoustic domain (Liberman and Mattingly, 1985; Fowler, 1986). In this view, for example, the neural circuits for speech sounds are tuned for vocal tract gesture and hardly respond to the change of acoustic sound itself. According to the second theoretical stance, listeners perceive articulatory movements relatively invariant to acoustic changes, instead of acoustic features. In short, it is likely that speaking and listening are tightly coupled with each other and both are regulated by the same structural constraints and grammar.

In the same context, we have already found that speech codes can be differentially generated and maintained in distinct neural circuits, according to whether the incoming acoustic waves are perceived as meaningful sounds or not (Yoo et al., 2012). We introduced novel sounds with an ambiguous vowel sound. The sounds could be perceived as either a word or a pseudoword according to the interpretation of the vowel. In this way, we could examine how higher linguistic factor modulates speech codes while the acoustic features of speech sounds were not changed. Interestingly, the perception of meaningless sounds (pseudowords) was supported by articulatory codes separately reserved in left inferior frontal gyrus (LIFG). It implies that before learning, speech perception might be supported by articulatory circuits for movement imitation (Iacoboni, 2005; Iacoboni and Dapretto, 2006). Furthermore, if it were the case, articulation or motoric movements are likely to have a certain role in perceiving sounds other than speech.

In our previous study, neural activities were modeled as vascular response observed by functional MRI, i.e., cerebral blood oxygenation (Blood Oxygenation Level Dependent, BOLD) followed by hemodynamic activities. The neuronal activation causes metabolic changes, and as a result, the amount of deoxy-hemoglobin (HHb) also changes. As HHb is paramagnetic, this change is observed in T_2_ weighted MRI. However, the BOLD contrast is known as a complex function of cerebral blood flow (CBF), cerebral blood volume (CBV), cerebral metabolic rate of oxygen (CMRO_2_), and so on. To describe neural activities more exactly, therefore, we need to measure these parameters independently and investigate how they interact with each other.

Currently, CBF can be measured by perfusion MRI, e.g., arterial spin labeling (ASL) MRI. While BOLD signal reflects changes in local HHb, CBF measured by perfusion MRI indicates the rate of delivery of metabolic substrates. For this reason, regional change of CBF (rCBF) is closer to neural activity than that of BOLD. However, it is less sensitive than BOLD and has lower temporal resolution same to fMRI. For CBV measurement, bolus injection is usually introduced. If we measure both CBF and CBV independently, we can estimate CMRO_2_, indicating that we can specify neural activities in a comprehensive way. However, the bolus injection is not practical to be used widely in that it is invasive.

As an alternative to the above, we considered functional near-infrared spectroscopy (fNIRS). The CBV is known to be in proportion to total hemoglobin change (Takahashi et al., 1999), and relative CMRO_2_ is positively correlated with CBF and oxygen saturation (StO_2_) (Watzman et al., 2000). The StO_2_ is measured by the proportional change of O_2_Hb over total Hb. This means that fNIRS is a simple way to provide both CBV and StO2 by measuring O_2_Hb and HHb with a high temporal resolution. It is possible to observe CBF in fNIRS (Elwell et al., 2005), but it sometimes requires injection of tracer. Instead, we can estimate it from the non-linear relationship (a constant power law) between CBF and CBV (Brown et al., 2003). It is not yet clearly known how CBF and CBV changes during neural activation, but it is reported that greater increases in CBF than in CBV are observed during neural activation (Ito et al., 2004).

In this study, based on these findings, we continued to investigate speech (or sound) processing at LIFG by observing O_2_Hb and HHb. Here, we designed more natural situation of speech communication, i.e., freely listening to various sounds and responding to speech sounds only. The experiment design is a bit similar to infants' word learning, in that they selectively mimic human speech sounds out of various environmental sounds. Contrasting time-varying regional difference of Hb concentration successfully provided us with how speech could be distinguished from other sounds, i.e., natural sounds, animal vocalizations, and human non-speech sounds. In addition, verbal repetition of words and pseudowords revealed how meaningful (words) and meaningless (pseudowords) speech are distinguished from each other in terms of Hb concentration changes.

## Materials and methods

### Subjects

Fifteen native Korean adults (9 males and 6 females) aged 19–37 years old (mean 25.3 years) participated voluntarily in this study. Informed consent was obtained from all participants before the experiment. The experiment procedure was approved by the Institutional Review Board of Seoul National University Hospital. All participants had normal auditory ability and reported no neurological deficits. The subjects completed a questionnaire to assess their handedness, according to the Edinburgh Handedness Inventory (Oldfield, 1971), and all were strongly right-handed (scored 80 or higher).

### Stimuli

The auditory stimuli were prepared in five different categories. They were classified into five categories according to their linguistic structures: (1) natural sounds, (2) animal vocalizations, (3) human non-speech sounds, (4) pseudowords, and (5) words (Table 1).

**Table 1:** **Classification of auditory stimuli**.

**Category**	**Linguistic meaning**	**Linguistic segment**	**Same species**	**Vocally produced**	**Sound**
Natural sounds	−	−	−	−	−
Animal vocalizations	−	−	−	+	+
Human non-speech sounds	−	−	+	+	+
Pseudowords	−	+	+	+	+
Words	+	+	+	+	+

The natural sounds were selected from the Pittsburgh Natural Sounds dataset recorded by Laboratory for Computational Perception and Statistical Learning (CNBC Lab., Carnegie Mellon University, USA). It consisted of ambient sounds (rain, wind, streams) with acoustic transients (snapping twigs, breaking wood, rock impacts) around the Pittsburgh region. Recording was carried out using a M-Audio's MobilePre-USB 16-bit/48 KS/s USB-powered Microphone Pre-amp, with all recordings made at 44,100 Hz. Twenty sound files out of the dataset were selected and then cut to be 2-s-length with normalized loudness as.*wav* files.

The animal vocalizations were collected from Avisoft Bioacoustics, Germany. It covered various animal vocalizations such as monkey, bird, sheep, horse, frog, etc. The recordings were made using SENNHEISER microphones K3/ME80, ME88, K6/ME62, 64, 66 or MKH60 connected to either a SONY DAT recorder TCD-D3, Marantz PMD 671, TASCAM DR-1, HD-P2, SONY PCM-M10, PCM-D50, or Fostex FR2-LE. We again selected twenty sound files from the data set: monkey (4 ea), sheep (1 ea), horse (1 ea), dog (4 ea), wolf (1 ea), mice (2 ea), birds (3 ea), frog (2 ea), and bat (2 ea). All files were cut to be 2-s-length and normalized as.*wav* files.

The human non-speech sounds were collected from the various web sites. We used twenty sound files, consisting of gasp (2 ea), giggle (2 ea), slurp (2 ea), burp (1 ea), cry (1 ea), yawn (2 ea), kiss (2 ea), slurp (2 ea), snore (2 ea), breathe (1 ea), scream (1 ea), and cough (2 ea). All were recorded as.*wav* files and normalized with the same length (2 s) in duration.

The pseudowords were generated by randomly combining several consonants and a vowel (/*a*/) in Korean, and thus have no meaning in Yonsei Korean Corpus 1–9 (Yonsei Korean Dictionary, 1998). The words were selected from the same Corpus, with balanced word frequency. All pseudowords and words were four syllable lengths. The pseudowords and words spoken by a female Korean native speaker were recorded and converted into computer files of.*wav* format (22,050 Hz, 16bit, stereo). The loudness (average RMS level) of all stimuli was normalized (−60 to 0 dB) by a sound software (SoundForge; Sony Creative Software Inc.).

All stimuli were not significantly different in loudness and did not exceed 2 s in total length. As shown in Table 1, the stimuli were classified in terms of several linguistic features, i.e., whether they have linguistic meaning, whether there is linguistic segment, whether they are produced by same species (aka human), whether they are vocally produced, and whether they are acoustic sounds.

### Experimental procedures

Lying in a comfortable table, the subjects were asked to repeat what they heard binaurally via an ear microphone in case of pseudowords and words, and otherwise simply listen to the stimuli. The sound volume was relevantly adjusted for comfortable and clear listening. In one category, twenty stimuli were used and totally 100 different stimuli in five different categories were presented to the subjects. The auditory stimuli in five different categories were pooled and then randomly presented to the subjects in four runs (twenty-five stimuli for each run).

**Figure 1:**
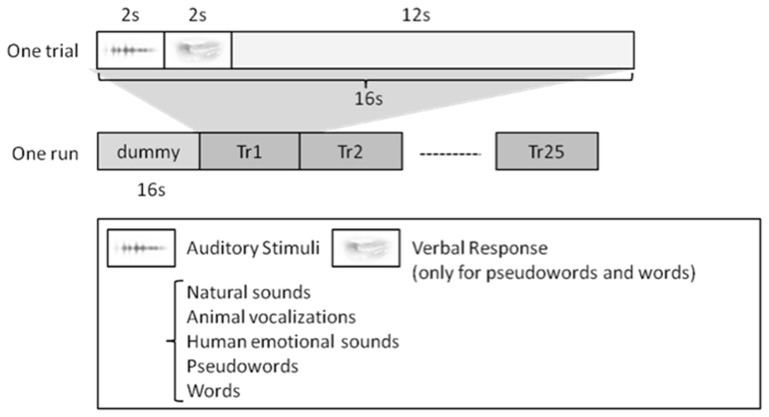
**Experiment Design.** For speech sounds, i.e., words and pseudowords, the subjects were asked to repeat what they heard. For the other stimuli, i.e., natural sounds, animal vocalizations, and human non-speech sounds, they simply listened to the stimuli. One trial lasted 16 s in length, and each run consisted of 25 trials. Each subject had four separate runs.

One trial consisted of 2 s of perception, 2 s of production (only for pseudowords and words), and 12 s of resting to avoid interference from other trials (Figure 1). Therefore, the length of one session was 416 s, including initial dummy 16 s (totally 6 min. 56 s.). Note that there was no production phase for natural sounds, animal vocalizations, and human non-speech sounds.

### Data acquisition

During the tasks, the hemodynamic changes at inferior frontal gyrus (LIFG) were bilaterally monitored by functional near-infrared spectroscopy (fNIRS). The LIFG was identified as the locus of articulatory code recruited during verbal repetition (Yoo et al., 2012), and its right homologue was selected as an experimental control. We used Oxymon Mark III 8-channel system with sampling rate of 250 Hz (Artinis, The Netherlands), which was capable of measuring the oxygenated (O_2_Hb) and deoxygenated (HHb) hemoglobin concentration changes of the optical paths (banana-shaped) in the brain between the nearest pairs of transmitter and receiver.

**Figure 2:**
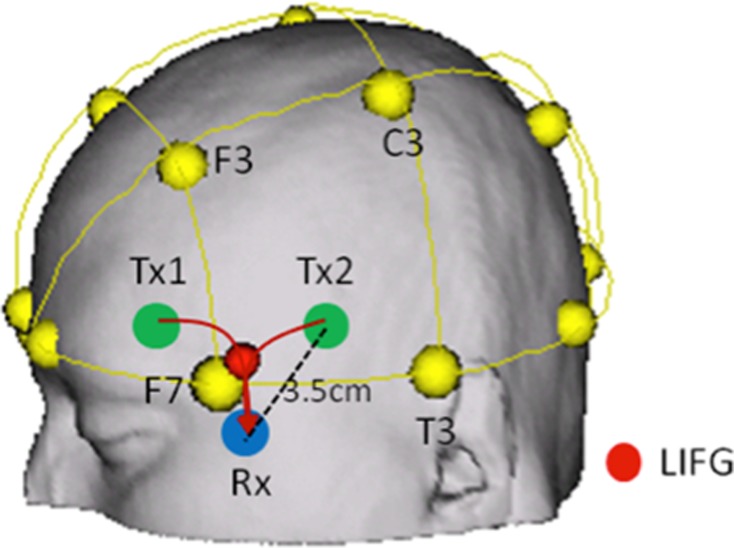
**Locus for NIRS monitoring (only left side was shown here).** To detect Hb concentration changes at IFG, we positioned one receiver and two transmitter optodes near IFG (BA47) bilaterally. The transmitter and receiver were separated by 3.5cm from each other. The travelling pathways of light are determined by the distance between transmitter and receiver, source wavelengths, characteristics of medium (tissue), and so on. The detecting depth was relevantly corrected to focus on the deep gray matter in inferior frontal gyri.

The NIRS emits 2 wavelengths (763 and 860 nm) of continuous near-infrared lasers. We introduced a 4 × 1 configuration for measuring hemodynamic change (Figure 2), each of which was modulated at different frequencies to detect O_2_Hb and HHb at two different brain areas, i.e. left and right inferior frontal gyri (BA47). The one of activated locus (LIFG, [−22 18 −22]) in Yoo et al. (2012) and its right homologue were selected as translated into a coordinate of the 10-20 system (10/20 [−1.9 0.87]) on the scalp surface by Münster T2T-Converter (NRW Research Group for Hemispheric Specialization, Münster University). As a part of LIFG, the locus was selected to measure more stable NIRS signals with relatively high SNR bilaterally. In addition, we obtained two regionally-separated signals in left and right inferior frontal gyri and compared the results. This could make it clearer to interpret the experimental result.

To detect the hemoglobin concentration changes at the loci, we separated the distance between transmitter and receiver by 3.5 cm on the scalp surface (Figure 2), and used differential path length factor (DPF) of 4, by which we could measure hemodynamic changes in the gray matter on the inner brain (Fukui et al., 2003). Using the modified Beer-Lambert law (Cope and Delpy, 1988), we calculated the concentration changes of oxy- and deoxygenated hemoglobin. In this study, it is difficult to calculate exactly cerebral blood flow and blood volume from the oxy− and deoxygenated hemoglobin concentration because we did not have all required parameters to calculate them. However, we can safely assume that the cerebral blood flow (CBF) is largely correlated with the concentration change of oxygenated hemoglobin, whereas the cerebral blood volume (CBV) are equally correlated with oxy- and deoxygenated hemoglobin changes (Lammertsma et al., 1984; Edwards et al., 1988). Based on this assumption, we interpreted the experimental results.

### Data analysis

The acquired data were analyzed by the followings: at four optode sites, the NIRS system provided oxygenated (O_2_Hb) and deoxygenated (HHb) hemoglobin concentration calculated by the modified Beer-Lambert law (Cope and Delpy, 1988). In one session, the time-series signals at one optode consisted of twenty-five trials randomly selected from five different conditions. For a subject, we collected one hundred trials (20 trials × 5 conditions) across four different sessions. The collected time-varying signals (2 signals × 4 optodes) were low-pass filtered with cutoff frequency of 10 Hz (5^th^-order Butterworth filter) to remove high frequency noises and motion artifacts. As we aimed to see the difference of neural responses between left and right IFG, the signals (O_2_Hb and HHb) at two ipsilateral optodes were averaged in the same hemisphere to obtain higher signal-to-noise ratio (SNR). Accordingly, we obtained time-varying data consisting of 2 signals × 2 hemispheres for a subject.

Then, O_2_Hb and HHb signals at left and right IFG were aligned at stimulus-onset-time to obtain event-locked response. From this, we could calculate hemodynamic response function (HRF) in this study. The HRF was simply estimated by averaging all event-locked trials in five different conditions for O2Hb and HHb signals, respectively. Considering peak timing difference between categories, there was a bit jittering. We discovered that the time courses of our HRFs to auditory stimuli had peaks between 5 and 6 s after the stimulus onset, which was comparable to the canonical HRF specified in most fMRI studies (Friston et al., 1995). It implies that the hemodynamic responses observed in this study are reliable enough to be used as an indicator for neural activities. Therefore, we assumed that the acquired data was suitable for further analysis.

Next, we divided the O_2_Hb and HHb signals at left and right hemispheres into five different categories. We averaged twenty, event-locked word-trials at each hemisphere and made a single, filtered time-point response for word repetition. The same averaging process was applied to twenty, event-locked pseudoword-trials. Similarly, we obtained the averaged neural responses for natural sounds, animal vocalizations, and human non-speech sounds at each optode, respectively. As there were two measures (O_2_Hb and HHb) at left and right hemispheres, we have twenty event-locked temporal responses by a subject (2 Hb measures × 2 loci × 5 categories).

Before statistical analysis, we calculated the proportional change of O_2_Hb over total Hb (sum of O_2_Hb and HHb), which is thought to be correlated to the CBF change. As a statistical analysis of these data, we first contrasted the proportional change of O_2_Hb over total Hb within two speech sounds, i.e., words and pseudowords across fifteen subjects. Considering the hemodynamic delay of neural responses (5–6 s after stimulus onset), we used time-binned signals including the peaks of HRFs, i.e. from 4 to 7 s (bin size = 3 s). The difference between words and pseudowords was confirmed by analysis-of-variance (Two-Way ANOVA), in which two independent variables were categories and measured optode sites. Within non-speech sounds, we contrasted the proportional change of O_2_Hb over total Hb in a similar manner.

As an indicator of CBV, the total Hb change was calculated from the averaged time-point responses of O_2_Hb and HHb for each category. Since the amount of total Hb change was so variable according to categories, we normalized it by subtracting the average of the total Hb and then divided it by its variance. In this way, the maximum value was adjusted to be less than two for all categories and the dynamic range of total Hb change was equal among all categories. With the normalized signals collected from fifteen subjects, we again conducted statistical analysis (Two-Way ANOVA) within speech sounds (2 categories × 2 loci). The same statistical analysis was applied to the normalized signals within non-speech sounds.

Lastly, we wanted to contrast the total Hb change of speech with that of non-speech. To this end, we calculated the averaged total Hb (HbT) change of words and pseudowords, and calculated the HbT change of non-speech sounds (natural sounds, animal vocalizations, and human non-speech). As explained in the above, we normalized them before statistical analysis. Across fifteen subjects, we conducted Two-Way ANOVA (2 categories × 2 loci). The significance of statistical analysis was all confirmed at α = 0.05.

## Results

**Figure 3:**
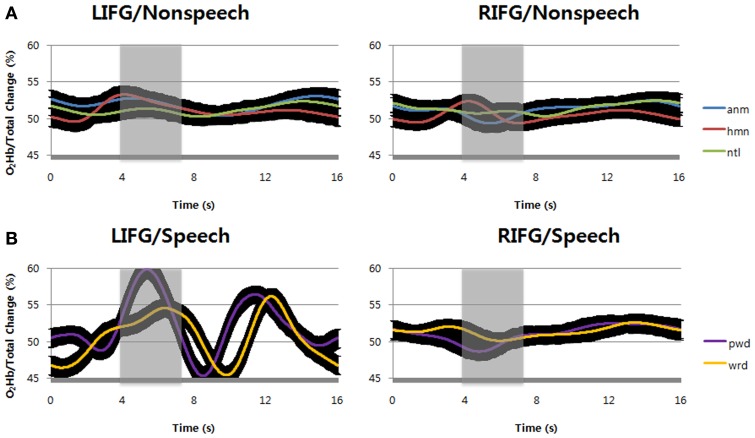
**(A)** Compared with the value at stimulus onset, the percent change of O_2_Hb over total Hb concentration by passive listening of non-speech sounds, i.e., animal vocalizations, human non-speech sounds, and natural sounds were shown. **(B)** The percent change of O_2_Hb over total Hb concentration by verbal repetition of speech sounds, i.e., words and pseudowords were shown. The black regions surrounding the signals show standard deviation. LIFG, left inferior frontal gyrus; RIFG, right inferior frontal gyrus; anm, animal vocalizations; hmn, human non-speech sounds; ntl, natural sounds; wrd, words; pwd, pseudowords.

We first examined the hemodynamic responses by repeating speech (pseudowords and words) and listening to non-speech sounds (natural sounds, animal vocalizations, and human non-speech sounds) at inferior frontal gyri (IFG, BA47) bilaterally during the tasks. Apparently, the result shows that speech sounds evoked higher hemodynamic responses at left inferior frontal gyrus than at right homologue, in terms of the percent change of O_2_Hb concentration over total Hb change (Figure 3B). In contrast to speech, non-speech sounds showed relatively small hemodynamic responses at the same locus (Figure 3A). At right inferior frontal gyrus, however, we found little hemodynamic responses for either speech or non-speech sounds. Among the stimuli tested in the current study, it is likely that only speech sounds can evoke a regional increase in cerebral blood flow at the left inferior frontal gyrus.

To support the above observation, we statistically analyzed the result and confirmed the difference between the types of stimuli and loci. In hemodynamic responses for speech sounds, it is notable that the O_2_Hb concentration changes by pseudoword repetition were higher than those by word repetition during the time windows of 4–7 s (shaded areas) after the stimulus onset (Figure 3B). It is reminiscent of the functional MRI study that left inferior frontal gyrus is reserved for articulatory speech codes of pseudowords (Yoo et al., 2012). The result was statistically significant at α = 0.05 [*F*_(1, 14)_ = 5.95, *p* = 0.0162; at 4–7 s time windows]. Consistently, its shape is more similar to the canonical HRF at the left inferior frontal gyrus (Friston et al., 1995), indicating that repeating pseudowords might recruit more neural circuits in this region. This result also indicates that there were large blood supplies with O_2_Hb to compensate O_2_ consumption by neural activities. In contrast, compared with the value at stimulus onset, we found no such Hb concentration change at the right inferior frontal gyrus for either words or pseudowords.

For non-speech sounds, compared with the value at stimulus onset, the O_2_Hb change over total Hb concentration was not found at either left or right inferior frontal gyrus (Figure 3A). In comparison with time windows of 1–4 s (before the shaded areas), the hemodynamic responses in shaded areas (time window of 4–7 s after the stimulus onset) were not significantly different at α = 0.05 [*F*_(2, 14)_ = 2.13, *p* = 0.1501 for left hemisphere; *F*_(2, 14)_ = 0.19, *p* = 0.6627 for left hemisphere]. In addition, for non-speech sounds, no main effects by either sound types or optode positions, and no interaction between them were found at α = 0.05 for the same time windows of 4–7 s (shaded areas) after the stimulus onset [*F*_(2, 14)_ = 1.13, *p* = 0.2901 for sound types; *F*_(2, 14)_ = 0.6, *p* = 0.5508 for optodes; *F*_(2, 14)_ = 0.6, *p* = 0.5494 for interaction].

**Figure 4:**
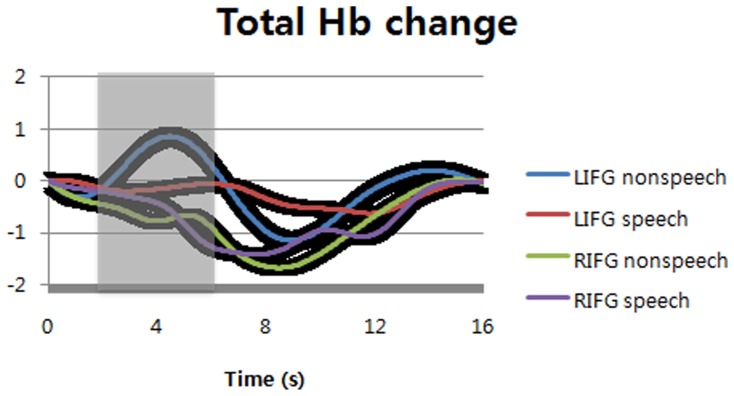
**Total hemoglobin changes at bilateral inferior frontal gyri (normalized as the scale of canonical hemodynamic response function with a range from 0 to 1).** The black regions surrounding the signals show standard deviation. LIFG, left inferior frontal gyrus; RIFG, right inferior frontal gyrus.

Interestingly, non-speech sounds could increase total Hb concentration at left inferior frontal gyrus while speech sounds could not change total Hb concentration (Figure 4). At right inferior frontal gyrus, both speech and non-speech significantly decreased total Hb concentration. The difference between speech and non-speech at left inferior frontal gyrus was significant during the time windows of 2–6 s (shaded areas) after the stimulus onset [*F*_(1, 14)_ = 6.58, *p* = 0.012 for sound types; *F*_(1, 14)_ = 4.12, *p* = 0.0447 for optodes; *F*_(1, 14)_ = 0.57, *p* = 0.4536 for interaction]. The time-to-peak (TTP) of hemodynamic response function is variable, i.e., about 5~6 s. In this study, we found the TTP of non-speech sounds was slightly less than that of speech sounds. For this reason, we shifted the time window of non-speech sounds for statistical analysis to make sure that the peak point is located in the middle of the comparison window.

Note that listening to non-speech sounds increased total Hb concentration at LIFG, whereas the same task could not change the O_2_Hb change over total Hb concentration at the same locus. For speech sounds, it was observed in opposite direction, i.e., speech sounds increased the O_2_Hb change over total Hb concentration, whereas the same sounds could not change total Hb concentration at LIFG. Interestingly, we found negative changes in both speech and non-speech sounds in terms of total Hb concentration, but there were little changes in terms of the O_2_Hb change over total Hb concentration for either speech or non-speech sounds. All these imply that regional CBV and CBF might be separated from each other by neural activities, which has time-varying characteristics. They are hardly separable from each other in fMRI measuring BOLD signals.

**Figure 5:**
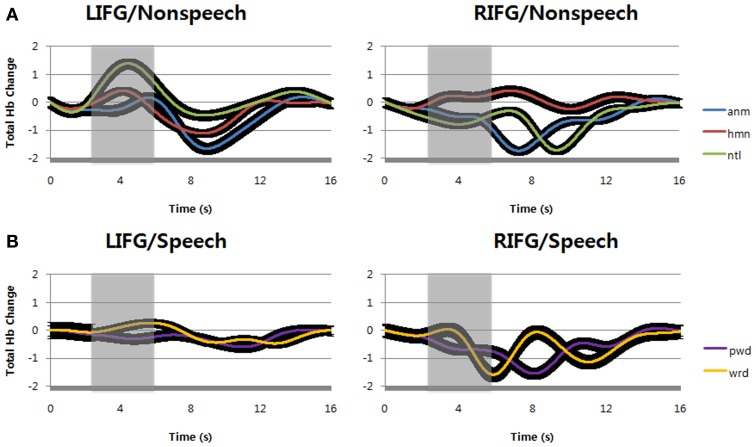
**(A)** Total hemoglobin changes by passive listening of animal vocalizations, human non-speech sounds, and natural sounds (normalized as the scale of canonical hemodynamic response function with a range from 0 to 1). **(B)** Total hemoglobin changes by verbal repetition of words and pseudowords (normalized). The black regions surrounding the signals show standard deviation. LIFG, left inferior frontal gyrus; RIFG, right inferior frontal gyrus; anm, animal vocalizations; hmn, human non-speech sounds; ntl, natural sounds; wrd, words; pwd, pseudowords.

During verbal repetition of words and pseudowords, we found little change of total Hb concentration at left inferior frontal gyrus (Figure 5B). Total Hb concentration was decreased for both words and pseudowords at right inferior frontal gyrus. The difference between words and pseudowords was not significant at either left or right inferior frontal gyri, and there was no interaction between sound types and optode positions during the time windows of 2–6 s (shaded areas) after the stimulus onset [*F*_(1, 14)_ = 1.78, *p* = 0.1849 for sound types; *F*_(1, 14)_ = 0.29, *p* = 0.5911 for optodes; *F*_(1, 14)_ = 0.35, *p* = 0.5525 for interaction].

In case of non-speech sounds, we observed increases of total Hb concentration by natural sounds at left inferior frontal gyrus (Figure 5A). Human non-speech sounds also evoked small increase of total Hb concentration at the same locus, but there was no change for animal vocalizations. At right inferior frontal gyrus, however, only human non-speech sounds could increase total Hb concentration while the other sounds decreased the changes of total Hb concentration. In other words, human non-speech sounds could increase the cerebral blood volume at bilateral inferior frontal gyri, implying the change of cerebral blood flow (Figure 3A).

This result implies that non-speech sounds can increase cerebral blood volume at left inferior frontal gyrus, but not at right inferior frontal gyrus. Statistical analyses showed that the difference between left and right inferior frontal gyri was significant at α = 0.05 during the time windows of 2–6 s (shaded areas) after stimulus onset time. However, there was no main effect of sound types, nor interaction between sound types and optodes [*F*_(2, 14)_ = 3.97, *p* = 0.0478 for sound types; *F*_(2, 14)_ = 1.8, *p* = 0.1687 for optodes; *F*_(2, 14)_ = 2.05, *p* = 0.1325 for interaction].

## Discussion

We examined hemodynamic responses at bilateral inferior frontal gyri (IFG, BA47) while the subjects verbally repeated speech sounds. We observed that the percent change of O_2_Hb concentration over total Hb was significantly higher for pseudowords than for words at left inferior frontal gyrus (LIFG). This result is consistent with the previous findings in Yoo et al. (2012). Interestingly, we also found significant increases of total hemoglobin concentration at LIFG even by passive listening of various non-speech sounds, which provides a new insight on sound perception in verbal repetition.

### Articulation-based code at left inferior frontal gyrus

The main purpose of this study was to re-examine the findings in Yoo et al. (2012), i.e., whether pseudowords are differentially represented in left inferior frontal gyrus (LIFG), in contrast to words. In the fMRI study, we suggested that unfamiliar speech sounds such as pseudowords might use articulatory codes based on sound imitation at the LIFG, and this was not the case in word repetition. In this context, we expected that the percent change of O_2_Hb concentration over total Hb at LIFG, similar to BOLD signal change in fMRI, would be significantly higher for pseudowords than for words. It implies that there were more changes in oxygen saturation level by neural activation, leading to abrupt increases in CBF to compensate this change (Watzman et al., 2000). The result was exactly replicated in this study.

The region investigated in this study is slightly displaced from the peak locus found in Yoo et al. (2012). Nevertheless, there were regional changes by repeating meaningless sounds. This means that the change of regional cerebral blood flow (rCBF) estimated by fNIRS conformed to the regional BOLD signal change measured by fMRI. It also implies that the LIFG were likely to be locally reserved as a temporal storage of speech codes for pseudowords during verbal repetition (Yoo et al., 2012). With respect to the result, it is notable that BA47 is known as a part of speech production circuits involved in fluency controls (Brown et al., 2005; Kell et al., 2009). This is partly consistent with the finding in this study, in which it will be critical to prepare articulatory codes for fluent speech before learning of pseudowords.

It is also notable that there were relatively small but considerable increases in O_2_Hb concentration by word repetition at the LIFG. In this case, it was likely that articulatory coding was automatically initiated at the LIFG while perceiving words. Unfortunately, due to the limitation of fNIRS channels in this study, we could not measure the O_2_Hb change at left middle temporal gyrus (LMTG), supposed to be a center of acoustic-phonetic codes of words (Yoo et al., 2012). According to our previous results, however, it is more likely that the acoustic-phonetic codes at the LMTG became superior to the articulation-based codes at the LIFG for words. That is, two distinct neural activities at the LIFG and LMTG seem to be simultaneously evoked for perceiving words.

This is partly because the LIFG serves as speech parser to detect word segmentation in continuous speech sounds (McNealy et al., 2006). McNealy and colleagues observed left-lateralized signal increases in temporal cortices only when parsing the continuous sounds with statistical regularities, which was a precursor of words. More importantly, they found that neural activities at LIFG and LMFG were positively correlated with an implicit detection of word boundaries, i.e., the detection of speech cues. That is, the LIFG might act as speech segmentation circuits automatically recruited before auditory lexical retrieval was completed at the LMTG (Marslen-Wilson, 1987).

On the other hand, the LIFG was known as a part of human mirror neuron system, supposed to be neural correlates of imitation mechanism (Iacoboni, 2005; Iacoboni and Dapretto, 2006). This notion is easily suited for the articulation-based sound perception discussed in the above, in that unfamiliar sounds are apt to be imitated for verbal repetition. In the same context, the O_2_Hb change by word repetition observed in 4–7 time windows at the LIFG was likely to be originated from the analysis-by-synthesis facility to perceive the incoming speech sounds (Cohen et al., 1988).

The higher response of pseudowords might be accounted for by other causes such as the difficulty to memorize and repeat pseudowords, compared to words. To be sure that the subjects clearly listen to the stimuli, we carefully adjusted the loudness of stimuli for each subject and minimized environmental noises during the task. No subjects reported listening problem of pseudowords in practices conducted before this experiment. The syllable length of pseudowords is four, same to the length of words, which is less than the capacity of verbal short-term memory (Miller, 1956). Therefore, we assume that pseudowords are not more difficult to repeat than words in terms of syllable length. The repeating time is at 2 s after listening to the stimuli, which is surely in the order of the duration of verbal short-term memory.

Another possibility is that novelty in pseudowords might enhance the hemodynamic response for pseudowords. Human brain can detect novel events at sub-cortical level by encoding regularities in the recent auditory past (Slabu et al., 2012), but the pseudowords used in this study were not novel in this sense because each syllable in pseudowords was a proper syllable currently used in Korean. At cortical level, articulating pseudowords might evoke novelty effects in the mind because there is no corresponding mental lexicon for the sounds. That is, at this level, the novelty is introduced by generating articulatory codes and this is exactly what we expected in this study (Yoo et al., 2012).

Lastly, notice that the second positive peak is observed in word and pseudoword repetition commonly (Figure 3B). The peak of pseudoword repetition was found at about 11.39 s after the stimulus onset, followed by that of word repetition at about 12.28 s after the stimulus onset. The second peaks seem to reflect the speech production after listening to the sounds. Consistent with the notion, no second peaks were found in non-speech sounds because the subjects passively listened to non-speech sounds without verbal repetition of perceived sounds. The small phase difference of second peaks between words and pseudowords might be due to the difference of preceding events for perception.

### Percent O_2_Hb change vs. total Hb change

It is interesting that no peaks were found in the proportions of O_2_Hb change over total Hb concentration for non-speech sounds despite that we found significant O_2_Hb change at the LIFG (Figure 3). In terms of total Hb change, however, non-speech sounds evoked large peaks at the LIFG while speech sounds did not change total Hb concentration (Figure 4). Therefore, it seems that total Hb change as well as percent O_2_Hb change is important to describe neural activities, indicating that there is a strong non-linear relationship between neural activity and hemodynamic response (Brown et al., 2003).

We can hardly observe the above finding in BOLD-fMRI. The BOLD-fMRI can measure more CBV-related changes, whereas the fNIRS can estimate both CBV and CBF by measuring hemodynamic changes of HHb and O2Hb at the same time. In practice, the BOLD-fMRI is likely to have more artifacts indicated as neural activities than ASL-fMRI measuring regional changes in CBF despite that they have a high congruency in activated patterns (Kemeny et al., 2005). The main reason why the BOLD-fMRI overestimates neural activities is because BOLD contrast is a result of neurovascular coupling determined by lots of physiological events, e.g., blood oxygenation, cerebral blood flow (CBF), and cerebral blood volume (CBV) (Buxton et al., 1998; Logothetis, 2002).

To overcome this technical limitation, it is highly required that fMRI methods based on BOLD contrast are used in combination with other methods, e.g., ASL-fMRI to examine changes in blood oxygenation and CBF (Detre and Wang, 2002). Multi-modal imaging is also helpful to overcome a spatial and temporal limitation in measurement. However, multi-modal imaging is usually very complex and not cost-effective. Huppert and his colleagues showed that temporal dynamics of BOLD response were well correlated with the NIRS measure of HHb, indicating that fNIRS may be used as an alternative of fMRI (Huppert et al., 2006). In addition, the fNIRS can estimate cerebral metabolic rate to separate CBV and CBF (Boas et al., 2003).

The total Hb change measured in fNIRS is generally thought to reflect the change of regional cerebral blood volume (rCBV), i.e., in proportion to rCBV (Villringer and Chance, 1997; Takahashi et al., 1999). In this study, we found that the total Hb increased only in listening to non-speech sounds at LIFG, compared to speech sounds (Figure 4). This is a replication of our previous study using BOLD-fMRI in that BOLD-fMRI tends to reflect a regional change of CBV. That is, neural circuits for non-speech sounds are subserved by the change of rCBV rather than that of rCBF. It thus seems that rCBV is more important to generate and maintain articulatory codes for non-speech sounds (Yoo et al., 2012).

This abrupt change of rCBV at LIFG is not observed in speech sounds. Instead, the percent Hb change over total Hb change was significantly high for speech sounds (Figure 3). The percent Hb change over total Hb concentration is positively correlated with cerebral metabolic rate of oxygen consumption (CMRO_2_), and both rCBF and CMRO2 are coupled with each other during cognitive tasks (Hoge et al., 1999; Watzman et al., 2000). It means that repeating speech sounds evokes more rCBF change than repeating non-speech sounds at LIFG.

The fMRI cannot discriminate the increase of rCBF from that of rCBV because the increase of rCBF is followed not only by neural activation, but also by cerebral vasodilation at systolic phase of the cardiac cycle (Lerch et al., 2012). However, the relation between changes in rCBF and rCBV seems to be de-coupled during neural deactivation, indicating that there might be a different mechanism between them (Ito et al., 2004). It suggests that speech and non-speech sounds are differentially processed in neural circuits at LIFG during deactivated phase. In addition, discordant responses to rCBF and rCBV are often reported in pharmacological MRI (Luo et al., 2009). It also suggests that neural circuits at LIFG operate differentially for both speech and non-speech sounds in terms of oxygen metabolism.

### Sound perception at bilateral inferior frontal Gyri

Speech perception has been traditionally considered in a sensory or acoustic domain. Recently, however, some theories based on non-sensory domain are emerging to account for neural mechanism of speech perception. For example, the motor theory suggests that listener perceives not the acoustic features, but the abstract intended gestures required to articulate the sounds (Liberman and Mattingly, 1985). As another variant of the motor theory, direct realism tries to account for speech perception as perceiving actual vocal tract gestures using information in the acoustic signal (Fowler, 1986). These all presuppose that perceiving sounds intrinsically involves motoric movements (Fadiga et al., 2002).

After Broca's seminal discovery, the left inferior frontal gyrus (LIFG) was reported as the center of speech production of fluent and articulated speech as well as that of speech comprehension (Caramazza and Zurif, 1976). This means that speech perception is partly dependent on the LIFG. Our results further suggest that the LIFG might have a certain role in perceiving non-speech sounds, too. The non-speech sounds, e.g., natural sounds and animal vocalizations used in this study were not articulable in terms of human vocal organs. It is thus less likely that the subjects might subvocally articulate the non-speech sounds during passive listening. Nevertheless, there were significant hemodynamic changes by perception of non-speech sounds at the LIFG, which was comparable to speech sounds, in terms of total Hb change (Figures 4, 5A).

With regard to this, it is reported that stimulus expectancy can modulate inferior frontal gyrus in passive auditory perception (Osnes et al., 2012). It is still debatable whether the LIFG has an essential or simple modulatory role in auditory perception, but motoric involvement is at least important in top-down control of auditory perception such as emotional arousal (Scott et al., 2009). This notion is supported by various sensorimotor integration mechanisms, too (Wilson et al., 2004; Pulvermüller et al., 2006; Wilson and Iacoboni, 2006). In addition, neural activities at the LIFG can predict individual differences in perceptual learning of cochlear-implant patients (Eisner et al., 2010), indicating that learning of sound perception is partly dependent on the LIFG.

However, it is not easy to interpret the hemodynamic modulation by the sound types at the LIFG (Figure 5A). It is likely to reflect the degree of internally simulated articulation to perceive incoming sounds, but it is not clear. Nevertheless, it should be noted that human non-speech sounds uniquely modulated total Hb changes at bilateral IFG, unlike the other sounds. It is possible that both left and right inferior frontal gyri responded to emotional stimulus, and as a result, autonomous nervous system (ANS) was activated. The activated ANS might change blood pressure and flow. To investigate it further, we need to see the whole brain areas with more NIRS channels, which can specify whether the change comes from local or global hemodynamic response.

In the line of emotional processing views, Hoekert and her colleagues revealed that both left and right inferior frontal gyri were involved in the processing of emotional prosody in speech (Hoekert et al., 2010). Another study with patients in supranuclear palsy reported that gray matter atrophy in RIFG has significant correlations with voice emotion recognition and theory of mind deficits, indicating that RIFG is associated with prosodic auditory emotion recognition (Ghosh et al., 2012). That is, the bilateral changes of total Hb concentration by listening to human non-speech sounds seem to be partly due to non-speech process in speech perception.

Putting all together, articulatory circuits at LIFG are involved in sound and speech perception. An auditory-motor integration was likely to develop in parallel with cognitive demands to organize incoming sounds as perceptually meaningful elements (Westerman and Miranda, 2002; Kuhl, 2004). The auditory-motor integration is also essential in social communication transferring non-verbal emotional states of others (Warren et al., 2006). Therefore, the hemodynamic changes at the LIFG suggest that auditory perception is in part supported by motoric representation, namely articulation-based sound perception.

### Conflict of interest statement

The authors declare that the research was conducted in the absence of any commercial or financial relationships that could be construed as a potential conflict of interest.
